# Evaluating quality of care for patients with type 2 diabetes using electronic health record information in Mexico

**DOI:** 10.1186/1472-6947-12-50

**Published:** 2012-06-06

**Authors:** Ricardo Pérez-Cuevas, Svetlana V Doubova, Magdalena Suarez-Ortega, Michael Law, Aakanksha H Pande, Jorge Escobedo, Francisco Espinosa-Larrañaga, Dennis Ross-Degnan, Anita K Wagner

**Affiliations:** 1 Division of Social Protection and Health, Inter-American Development Bank, Washignton, USA; 2 Epidemiology and Health Services Research Unit CMN Siglo XXI, Mexican Institute of Social Security, México, DF, Mexico; 3 Centre for Health Services and Policy Research, School of Population and Public Health, The University of British Columbia, Vancouver, Canada; 4 Department of Population Medicine and WHO Collaborating Center in Pharmaceutical Policy, Harvard Medical School and Harvard Pilgrim Health Care Institute, Boston, MA, USA; 5 Unidad de Investigación en Epidemiología Clínica, Hospital Regional 1 Carlos MacGregor Sánchez Navarro, IMSS, México, DF, México; 6 División de Innovación de la Coordinación de Educación en Salud,IMSS, México, DF, Mexico

## Abstract

**Background:**

Several low and middle-income countries are implementing electronic health records (EHR). In the near future, EHRs could become an efficient tool to evaluate healthcare performance if appropriate indicators are developed. The aims of this study are: a) to develop quality of care indicators (QCIs) for type 2 diabetes (T2DM) in the Mexican Institute of Social Security (IMSS) health system; b) to determine the feasibility of constructing QCIs using the IMSS EHR data; and c) to evaluate the quality of care (QC) provided to IMSS patients with T2DM.

**Methods:**

We used a three-stage mixed methods approach: a) development of QCIs following the RAND-UCLA method; b) EHR data extraction and construction of indicators; c) QC evaluation using EHR data from 25,130 T2DM patients who received care in 2009.

**Results:**

We developed 18 QCIs, of which 14 were possible to construct using available EHR data. QCIs comprised both process of care and health outcomes. Several flaws in the EHR design and quality of data were identified. The indicators of process and outcomes of care suggested areas for improvement. For example, only 13.0% of patients were referred to an ophthalmologist; 3.9% received nutritional counseling; 63.2% of overweight/obese patients were prescribed metformin, and only 23% had HbA1c <7% (or plasma glucose ≤130 mg/dl).

**Conclusions:**

EHR data can be used to evaluate QC. The results identified both strengths and weaknesses in the electronic information system as well as in the process and outcomes of T2DM care at IMSS. This information can be used to guide targeted interventions to improve QC.

## Background

The worldwide prevalence of type 2 diabetes (T2DM) is on the rise, especially in low and middle-income countries (LMIC), reaching up to 14% of the adult population in Mexico [1], twice the current global prevalence [2]. T2DM poses considerable challenges to healthcare systems because it is among the leading causes for ambulatory care, hospital admissions and death [3]. In Mexico, quality of T2DM care has been suboptimal [4,5], health outcomes poor, [6] and costs of T2DM care and its complications are on the rise [7].

Healthcare for patients with diabetes has both individual and health system goals. At the individual level, the goal is to achieve the best possible health outcomes. At the health system level, the goal is to provide accessible, high quality, effective, and efficient care. Information about progression of the disease and processes of care for patients with T2DM over time is required to assess whether goals are being accomplished.

The electronic health record (EHR) is a tool that can provide necessary input for dynamic improvement of systems for chronic care by a) supporting clinical decisions at the point of care; b) providing routine data to evaluate the quality of care (QC); and c) facilitating longitudinal systems research and efficient information sharing for dynamic care improvement [8,9]. In publicly financed health care systems, EHR may also help improve transparency and accountability.

LMIC are beginning to use EHR [10]. Argentina, Costa Rica and Peru use EHR in circumscribed settings, such as individual clinics or hospitals, or for research purposes [11]. Mexico was the first Latin American country that introduced an EHR on a large-scale at the Mexican Institute of Social Security (IMSS). IMSS is a nation-wide institution providing social security and healthcare benefits to approximately 47% of the 112 million Mexican people. In 2003, IMSS began to introduce its EHR and evidence-based clinical guidelines to improve performance of its family medicine clinics [12]. The IMSS EHR became a key component of the institutional information system, which also includes administrative, member enrollment, pharmacy and other databases.

The IMSS EHR comprises information that could be used to improve the quality, efficiency and continuity of care; however, it has not been fully used for these purposes. Little is known about the quality of the data that are routinely captured and what relevant quality of care indicators (QCIs) can be constructed based on routine data.

T2DM is a complex and dynamic problem that requires a steady flow of information for healthcare providers to guide their clinical and managerial decision-making. The objectives of this project were: a) to develop QCIs for T2DM in the IMSS health system; b) to determine the feasibility of constructing QCIs using IMSS EHR data; and c) to evaluate the QC provided to IMSS patients with T2DM.

## Methods

### Design

We used data from four large family medicine clinics located in Mexico City with a catchment area of 585,536 people. Each clinic had between 15 and 30 physician offices, a laboratory, and a pharmacy.

IMSS’ EHR consists of several linked databases which include information on appointments; medical history; physical examination; clinical encounter notes; aspects of care and progress for patients with specific conditions (diabetes, antenatal care, hypertension); social work, occupational health, dietary, and preventive care services. Clinical encounter notes capture the reason for the visit, symptoms, signs, diagnoses (ICD-10 coded), orders for laboratory tests, electronic prescriptions, and disability and referral information. Data fields contain free-text, numeric, or pre-coded, menu-driven entries. Electronic prescriptions (by menu-driven generic product name) are linked to the IMSS essential list of medicines and the clinic’s pharmacy inventory. The laboratory database is linked to the menu-driven, coded catalog of IMSS laboratory exams and contains laboratory test orders. Laboratory examination results reside in different, unlinked databases.

### Stage 1: Development of QC Indicators

To design and validate T2DM QCIs, we used the modified version of the RAND/UCLA Appropriateness Method, which comprises scientific evidence and expert opinion [13,14].

First, we performed a systematic search and review of the literature on QCIs for T2DM using Marshall’s proposed method [15]. We searched the following databases: Medline, Ovid, Cochrane Library, National Guideline Clearinghouse, CMA Infobase: Clinical Practice Guidelines, TRIP database, Institute for Clinical System Improvement, ACP Guideline website, American Academy of Family Physicians, NHS Evidence - National Library of Guidelines, and IMSS-Clinical Guidelines [16]. The keywords were “type 2 diabetes mellitus”, “quality of care indicators”, “clinical guidelines,” and “family medicine” or “primary care services,” “diagnosis” and “treatment”.

The review focused on evidence-based care processes shown to increase the likelihood of achieving the best possible clinical outcomes, following the criteria proposed by Saslow et al. [17]. The review included indicators from the Healthcare Effectiveness Data and Information Set (HEDIS) [18], as well as those developed by the RAND Health Research and Institute for Clinical System Improvement (ICSI) teams. Indicators for four basic components of care for T2DM patients were defined a) timely screening for complications and comorbidity; b) non-pharmacological treatment; c) pharmacological treatment; and d) health outcomes.

Next, we assembled a panel of 8 experts. Panel members had experience in treating T2DM patients, in conducting clinical and health systems research, and in developing clinical guidelines. Each panelist received information about the study objectives, methodology, literature review and set of preliminary indicators. Panelists rated indicators on validity and feasibility according to Shekell’s criteria [19] by assigning a value from 1 to 9 (1 = definitely not valid or not feasible and 9 = definitely valid or feasible). An indicator was valid when it assessed a process of care for which there was enough scientific evidence to support the relationship between the specific process and a potential health benefit or the assumption that health care professionals who perform specific care aspects provide better quality of care than those who do not. An indicator was feasible when there was a high probability that the required information to construct the indicator would be available in a typical health record, or, if not, the lack of information would represent poor quality of care. Panelists used these criteria to rate each proposed indicator. An indicator was valid and feasible if its mean score was ≥ 7 on each domain. Panelists participated in two e-mail rounds of ranking and two face-to-face panel meetings.

### Stage 2: Extraction of routine EHR data to construct the QCIs

To evaluate whether the indicators defined in Stage 1 could be constructed, we extracted data from a cohort of all continuously enrolled patients in the four study clinics. All patients with a diagnosis of T2DM according to the International Classification of Diseases, Tenth Revision (ICD-10) codes E111-E119, E140-E149, and E14X in one of three EHR diagnosis fields, who were older than 19 years of age and visited a family doctor at least once during 2009 were eligible.

For the cohort of patients with T2DM, we extracted EHR and laboratory data from the participating Family Medicine Clinics (FMCs) for calendar year 2009 and created an analytic dataset with the following variables:

a. Variables from the EHR: general patient characteristics (age, sex, schooling, marital status, membership (subscriber/dependent and employment status); medical history (age at onset and duration of T2DM, history of hypertension, other cardiovascular diseases, or dyslipidemia; chronic complications [peripheral vascular disease, nephropathy, retinopathy, and peripheral neuropathy]; physical measurements (weight, height, and blood pressure); nutritional status (at the beginning and at the end of the year) as measured by body mass index (BMI) (underweight [BMI <18.5 kg/m^2^], normal weight [BMI 18.5 - 24.9 kg/m2], overweight [BMI 25.0 - 29.9 kg/m^2^], obese [BMI ≥30.0 kg/m^2^]); clinical care (number of visits to the family doctor, referral to an ophthalmologist and/or dietitian); prescriptions (insulin and oral hypoglycemic drugs [metformin, glibenclamide, acarbose, rosiglitazone or pioglitazone]; other drugs [acetylsalicylic acid, statins and angiotensin converting enzyme inhibitors or angiotensin-receptor blockers]); laboratory tests ordered.

b. Variables from the laboratory database: dates and measured values of hemoglobin A1c (HbA1c), fasting blood glucose, and total cholesterol.

We queried the relational EHR and laboratory database tables of each clinic using standard queries in structured query language (SQL) to retrieve data from tables and create a new analytic database. We then assessed the data for completeness, consistency and accuracy. We predefined non-plausible lowest values for the following variables: blood pressure (systolic blood pressure <50 mmHg or >250 mmHg and diastolic blood pressure <40 mm Hg or >200 mmHg [20]); height (<130 cm or >250 cm), weight (<30 kg [21] or >200 kg), HbA1C (<3.0), fasting blood glucose (<37 mg/dl); and total cholesterol (<100 mg/dl). All non-plausible values were excluded from the analysis. We used the SAS statistical package (V9.2) to construct complex variables from the extracted data. We validated the extracted data by comparing a sample of 80 records in the EHR system with our final dataset. The comparisons were made reviewing data from individual EHR with the extracted data.

### Stage 3: Evaluation of T2DM quality of care

We described QC indicators by family medicine clinic. We also characterized clinics by available health personnel (number of medical doctors, nutritionists, social workers, and nurses) and number of examination rooms.

The IMSS’ National Commissions of Research and Ethics reviewed and approved the study protocol.

## Results

### Stage 1: Development of QC Indicators

Table 1 shows the results of the indicator evaluation process. Scores ranged from 5.3 to 8.7. Based on the predefined threshold of 7, the panel discarded 5 and accepted 18 indicators; some indicators scored high in validity, but were not feasible and were then modified. For example, HbA1c test results were not available in all clinics. Therefore, the outcome indicator was modified from “T2DM patients who had HbA1c <7%” to: “T2DM patients who had HbA1c <7%, or fasting blood glucose ≤130 mg/dl in the last three measurements”. Similarly, the LDL-cholesterol indicator was replaced by an indicator based on total cholesterol, while the indicator of screening for microalbuminuria was discarded.

**Table 1:** Results from evaluation of candidate indicators

**Indicator**	**Validity**	**Feasibility**	**Result of the validation process**	**Programmable in the EHR**
**I. Process indicators**	**Average**			
**A. Timely detection of T2D complications and comorbidity in the last year**				
1. At least one measurement of HbA1c	8.7	7.8	Accepted	Programmable
2. Comprehensive foot evaluation	8.7	8.2	Accepted	Programmable
3. Referral to the ophthalmologist	8.3	7.7	Accepted	Programmable
4. Screening for microalbuminuria through the ratio albumin/creatinine	8.5	5.7	Discarded	
5. Measurement of creatinine and rate of glomerular filtration	8.7	5.3	Discarded	
6. Screening for dyslipidemia by measuring total cholesterol in patients without previous diagnosis of dyslipidemia	8.5	7.8	Accepted	Programmable
**B. Non pharmacological treatment in the last year**				
7. Smoking cessation counseling for current smokers	8.2	8	Accepted	Not Programmable
8. Advise to moderate alcohol consumption	6.0	5.8	Discarded	
9. Nutritional counseling provided by the nutrition service	7.8	7.8	Accepted	Programmable
10. Advise to practice aerobic physical exercise of moderate intensity, at least 150 minutes per week, unless contraindicated	8.2	8.0	Accepted	Not Programmable
11. Registration of adherence to dietary recommendations	8.5	7.0	Accepted	Not Programmable
12. Registration of adherence to aerobic physical exercise	8.5	7.0	Accepted	Not Programmable
**C. Pharmacological treatment in the last three visits**				
13. Overweight/obese (BMI ≥ 25 kg/m^2^) patients who received metformin, unless contraindicated*	8.7	8.5	Accepted	Programmable
14. Patients with HbA1c ≥ 8, or with an average fasting blood glucose of ≥140 mg/dl in the last 3 months, who had registered: a) recommendations to modify their diet and physical exercise, b) referral to a social work group; c) modification in their scheme of treatment.	8.7	6.3	Discarded	
15. Patients with HbA1c ≥ 8, or with an average fasting blood glucose of ≥140 mg/dl in the last 3 months, who had registered adherence to the pharmacological treatment.	8.5	6.8	Discarded	
16. Patients with hypertension receiving inhibitors of angiotensin converting enzyme or angiotensin-receptor blocker, otherwise contraindicated**	8.5	8.5	Accepted	Programmable
17. Patients > 40 years of age with one or more of the following risk factors: smoking, hypertension, dyslipidemia, receiving 75-150 mg/day of acetylsalicylic acid, unless contraindicated***	8.5	8.2	Accepted	Programmable
18. Patient with total cholesterol >200 mg/dl and were prescribed statins, unless contraindicated****	8.2	8.3	Accepted	Programmable
**II. Health outcomes indicators**				
19. HbA1c <7% or fasting glucose ≤130 mg/dl in the last 3 measurements	8. 7	8.3	Accepted	Programmable
20. Total cholesterol levels < 200 mg/dl in the last measurement	8. 7	8.2	Accepted	Programmable
21. Blood pressure <130/80 mmHg in the last 3 measurements	8. 7	8.5	Accepted	Programmable
22. Overweight/obese (BMI ≥ 25 kg/m^2^) patients who lost ≥5% body weight in the last year	8.0	7. 7	Accepted	Programmable
**Composed indicator of health outcomes**				
23. Patients with HbA1c <7%, or fasting glucose ≤130 mg/dl, total cholesterol levels < 200 mg/dl and blood pressure <130/80 mmHg in the last 3 measurements	8.5	7.5	Accepted	Programmable

*Contraindications to metformin: a) Renal failure b) respiratory or advanced liver failure c) congestive heart failure, coronary artery disease or advanced atherosclerosis; d) pregnancy; e) intolerance to metformin.

**Contraindications to inhibitors of angiotensin converting enzyme: intolerance and/or prior treatment failure.

***Contraindications to acetylsalicylic acid in doses of 75-150 mg/day: history of hypersensitivity to aspirin, peptic ulcer disease, and hemophilia.

****Contraindications to statins: hypersensitivity to any component of the drug, active liver disease or unexplained persistent elevations of serum transaminases, pregnancy and lactation.

### Stage 2: Extraction of routine EHR data to construct QCIs

The final list (Table 1) shows that 14 indicators were programmable with data from the EHR and laboratory databases. We decided that four indicators could not be reliably constructed because information was only available in free-text fields with wide variability in entries. There was a high percentage of missing data for employment, schooling, marital status, and duration of diabetes. Also, weight, height and blood pressure had between 5% and 24% non-plausible values.

### Stage 3: Evaluation of T2DM quality of care

Table 2 presents the main characteristics of the four participating FMCs. There were 204 family doctors working in the clinics; 67 registered nurses, 86 auxiliary nurses, 6 dietitians, and 36 social workers. Three clinics had a separate department to conduct medical education activities. In 2009, the clinics covered 585,536 members and dependents, with 437,417 >20 years old; approximately 174,266 patients visited the clinics at least once during 2009, of whom 25,130 (14% of patients) had a diagnosis of T2DM.

**Table 2:** Population and clinic characteristics

**Population affiliated with the family medicine clinic**	**FMC A**	**FMC B**	**FMC C**	**FMC D**	**Total**
	**n**	**n**	**n**	**n**	**n**
Total number of members	123,276	149,396	196,513	116,351	585,536
Members per family doctor	2,241	2,449	3,388	3,878	2, 828
Members ≥ 20 years old	95,303	120,579	143,796	77,739	437,417
Members ≥ 20 years old who attended the clinic at least once in 2009	45,703	53,370	46,270	28,923	174,266
T2DM patients as percentage of members ≥20 years old who attended the clinic at least once in 2009	7184 15.7**%**	6671 12.5**%**	7256 15.7**%**	4019 13.9**%**	25130 14.4**%**
**Health personnel and examining rooms**					
Family doctors	55	61	58	30	204
Registered nurses	14	26	14	13	67
Ancillary nurses	14	21	35	16	86
Dietitian	2	1	1	2	6
Social workers	9	11	9	7	36
Number of family doctors’ offices	27	29	30	15	101
Department of medical education	1	1	1	0	3

Source: Family medicine clinics 2009 electronic health record information.

Table 3 presents the characteristics of the T2DM patients. Most (59%) were women. The average age was 62.3 years. Nearly two in five patients had low education (39% illiterate or primary school education only); almost half were married or lived with their partner. Nearly 30% were housewives; 17% were employed and 10% were retired. Most (76%) patients were dependents of insured members.

**Table 3:** Type 2 diabetes patients’ general characteristics

**Characteristics**	**FMC A**	**FMC B**	**FMC C**	**FMC D**	**Total**
	**n = 7184%**	**n = 6671%**	**n = 7256%**	**n = 4019%**	**n = 25130%**
**Female sex**	59.3	57.8	58.6	58.2	58.5
**Age, years,** mean (standard deviation)	62.9 (12.9)	64.3 (12.8)	61.8 (12.8)	58.5 (12.4)	62.3 (12.9)
**Age groups**					
<30 years	0.6	0.3	0.7	0.9	0.6
30–39 years	3.4	3.1	4.0	5.2	3.8
40–49 years	11.2	9.7	12.6	16.8	12.1
50–59 years	23.8	21.0	24.4	31.2	24.4
60–69 years	28.3	29.6	29.5	26.0	28.6
≥70 years	32.7	36.3	28.8	19.9	30.5
**Schooling**					
Illiterate	16.1	7.5	14.9	9.5	12.4
Primary school	24.3	17.6	37.7	26.9	26.8
Secondary school	11.9	11.5	14.3	10.6	12.3
High school	14.8	17.5	10.4	5.3	12.7
University degree	8.0	12.4	4.3	1.3	7.0
Missing data	0.9	33.5	18.3	46.4	28.7
**Marital status**					
Married or partnership	48.3	39.8	54.5	38.0	46.2
Single or divorced	11.9	12.6	7.9	4.5	9.7
Widow	14.2	12.8	2.9	7.1	13.0
Missing data	25.6	34.7	22.3	50.4	31.0
**Employment status**					
Housewife	32.7	26.1	35.3	17.7	29.3
Employed	19.3	16.3	19.6	12.6	17.5
Unemployed	0.1	0.2	0.1	0.4	0.2
Retired	11.9	12.0	11.4	3.1	10.4
Missing data	36.0	45.4	33.6	66.2	42.6
**Insurance status**					
Subscriber	24.0	25.9	20.6	22.4	23.3
Dependent	76.0	74.1	79.4	77.6	76.7

Source: Family medicine clinics 2009 electronic health record information.

Table 4 lists the clinical conditions and health care characteristics of the included patients. More than 16% of those reporting had diabetes for over 15 years; however, disease duration was mostly missing (44%-77%). Hypertension was the most frequent comorbidity (60%). Approximately 31% of patients had a chronic complication, mostly peripheral vascular disease (14%) followed by diabetic nephropathy (11%). Only 15% had normal weight; 36% were overweight and 33% obese.

**Table 4:** Clinical conditions and health care characteristics

**Medical history**	**FMC A**	**FMC B**	**FMC C**	**FMC D**	**Total**
	**n = 7184 %**	**n = 6671 %**	**n = 7256 %**	**n = 4019 %**	**n = 25130 %**
**Duration of diabetes**					
<5 years	3.8	2.5	1.6	0.9	2.4
5–10 years	12.3	6.8	5.3	10.3	8.5
11–15 years	11.6	7.3	5.2	7.5	7.9
>15 years	27.5	14.8	10.6	11.1	16.6
Missing data	44.8	68.6	77.3	70.2	64.5
**Comorbidity and chronic complications**					
Hypertensive disease	63.3	68.0	59.2	44.5	60.4
Other cardio-vascular disease	11.2	13.2	6.1)	4.2	9.2
Hyperlipidemia	49.2	44.3	40.2	26.1	41.6
Diabetic chronic complications	29.9	29.5	38.1	27.0	31.7
**Type of chronic complication**					
Peripheral vascular disease	8.9	6.8	26.8	14.9	14.5
Diabetic nephropathy	14.5	10.4	8.1	8.2	10.6
Diabetic retinopathy	7.0	12.1	6.8	4.4	7.9
Peripheral neuropathy	5.6	6.0	3.6	4.3	4.9
**Nutritional status**					
**Nutritional status** at the end of the year					
Under weight (<18.5 kg/m^2^)	0.3	0.5	0.2	0.3	0.3
Normal weight (BMI18.5- 24.9 kg/m^2^)	16.4	17.9	14.2	13.5	15.7
Overweight (BMI 25.0 - 29.9 kg/m^2^)	35.8	35.4	36.3	36.2	35.9
Obesity (BMI ≥30.0 kg/m^2^)	33.9	31.7	35.3	34.2	33.8
Missing data	13.6	14.5	14.0	15.8	14.3
**Health care characteristics**					
**Number of visits of the patient to the family doctor,** mean (standard deviation)	8.5 (4.4)	9.6 (5.0)	8.7 (4.5)	7.9 (4.1)	8.8 (4.6)
**Hypoglycemic prescriptions**					
**Number of hypoglycemic prescriptions**					
None	20.5	18.8	14.1	9.9	16.5
1	37.1	40.9	32.3	34.0	36.2
2	38.1	37.2	43.0	46.9	40.7
≥3	4.3	3.1	10.5	9.1	6.5
**Type of hypoglycemic drugs**					
Metformin	57.2	62.3	65.3	67.1	37.5
Glibenclamide	51.8	46.9	58.7	61.7	45.9
Acarbose	3.3	3.2	12.5	9.7	7.0
Thiazolidinedione	0.0	0.0	1.5	0.0	0.4
Insulin	14.1	12.4	12.5	17.4	13.7

Source: Family medicine clinics 2009 electronic health record information.

Patients with T2DM were regular users of health care services; on average, they had 8.8 visits in 2009. Between 10% and 20% of patients did not receive any hypoglycemic prescription; most (77%) received 1 or 2 drugs. Metformin (37%) and glibenclamide (46%) were the most frequently prescribed medications.

Table 5 reports results for the quality of care indicators. There were wide variations among clinics. Two clinics had no HBA1c measurements, while the other two had limited availability. Indicators of timely detection of complications varied widely: between 5%-52% of patients received comprehensive foot evaluations; 5%-22% were referred to an ophthalmologist; and 46%-70% were screened for dyslipidemia. Only 4% received nutritional counseling. Across clinics, metformin was prescribed (63%) to a majority of overweight or obese patients; 57% of patients with hypertension were prescribed ACE inhibitors; 43% of patients with risk factors for cardiovascular events received prophylactic acetylsalicylic acid; and 48% of those with hypercholesterolemia received a statin.

**Table 5:** Indicators of quality of care

**Indicators**	**FMC A**	**FMC B**	**FMC C**	**FMC D**	**Total**
**I. Process of care**	**n = 7184 %**	**n = 6671 %**	**n = 7256 %**	**n = 4019 %**	**n = 25130 %**
**A. Timely detection of T2D complications and comorbidity in the last year**					
At least one measurement of HbA1c	9.0	16.9	Not available	Not available	7.1
Comprehensive foot evaluation	51.9	28.2	24.6	5.4	30.3
Referral to the ophthalmologist	22.2	14.2	7.0	5.4	13.0
Screening for dyslipidemia by measuring total cholesterol in patients without previous diagnosis of dyslipidemia	**3653**	**3714**	**4336**	**2971**	**14674**
	65.4	54.7	45.6	70.0	57.8
**B. Non-pharmacological treatment in the last year**	**n**	**n**	**n**	**n**	**n**
	**%**	**%**	**%**	**%**	**%**
Nutritional counseling provided by the nutrition service	**7184**	**6671**	**7256**	**4019**	**25130**
	1.8	5.5	4.9	3.0	3.9
**C. Pharmacological treatment in the last three visits**	**n**	**n**	**n**	**n**	**n**
	**%**	**%**	**%**	**%**	**%**
Overweight/obese (BMI ≥ 25 kg/m^2^) patients who received metformin, unless contraindicated	**5066**	**4437**	**5216**	**2840**	**17559**
	57.2	63.0	66.7	67.6	63.2
Patients with hypertension receiving inhibitors of angiotensin converting enzyme or angiotensin-receptor blocker, unless contraindicated	**4545**	**4536**	**4298**	**1787**	**15172**
	46.0	56.8	58.9	66.1	57.4
Patients > 40 years of age with one or more of the following risk factors: smoking, hypertension, dyslipidemia, receiving 75-150 mg/day of acetylsalicylic acid, unless contraindicated	**5242**	**5035**	**4904**	**2076**	**17257**
	45.8	32.0	46.2	58.9	43.4
Patient with total cholesterol >200 mg/dl and were prescribed statins, unless contraindicated	**2436**	**1998**	**2197**	**1233**	**7864**
	55.6	43.9	49.1	36.9	47.9
**II. Health outcomes**	**n**	**n**	**n**	**n**	**n**
	**%**	**%**	**%**	**%**	**%**
HbA1c <7% or fasting glucose ≤130 mg/dl in the last 3 measurements	**4644**	**3560**	**4563**	**2816**	**15583**
	23.0	32.5	19.2	17.9	23.1
Total cholesterol levels < 200 mg/dl in the last measurement	**5097**	**4168**	**4125**	**3008**	**16398**
	52.2	52.1	46.7	59.0	52.0
Blood pressure <130/80 mmHg in the last 3 measurements	**7088**	**6587**	**7247**	**4011**	**24933**
	12.3	8.5	14.1	5.7	10.8
Overweight/obese (BMI ≥ 25 kg/m2) patients who lost ≥5% body weight in the last year	**5066**	**4437**	**5216**	**2840**	**17559**
	13.4	14.7	12.6	12.5	13.3
Patients with HbA1c <7%, or fasting glucose ≤130 mg/dl, total cholesterol levels < 200 mg/dl and blood pressure <130/80 mmHg in the last 3 measurements	**4272**	**3123**	**3479**	**2516**	**13390**
	1.8	1.7	1.2	0.6	1.4

Source: Family medicine clinics information from 2009 electronic health record. The analysis included 12 months.

Approximately 62% of patients had registered results of blood glucose and total cholesterol tests during 2009; among those without this information, 22% did not have a glucose and cholesterol test orders and 16% had the laboratory order registered, but these patients did not attend to the laboratory. After comparing the characteristics of patients with and without laboratory tests, we did not find statistically significant differences regarding sex, age, literacy or occupation, or co-morbidity. Differences were observed in relation to the number of medical visits to a family doctor, as a higher proportion of patients without laboratory exams had only 1 or 2 visits per year (1.2% overall and 21.5% among patients without laboratory results) (Data not included in the table).

Among patients with laboratory exams, few had achieved desired health outcomes: 23% had HbA1c <7% or fasting glucose ≤130 mg/dl in the last 3 measurements; 52% had total cholesterol <200 mg/dl in the last measurement; 11% had blood pressure <130/80 mmHg in the last 3 measurements; 13% of overweight or obese patients had lost ≥5% body weight in the last year; and only 1% of patients reached the combined therapy goals for blood glucose, cholesterol and blood pressure control.

## Discussion

Our results suggest that it is feasible to a) develop T2DM QCIs applicable in the context of a middle-income country; b) measure the QCIs using routinely collected data from a widely implemented EHR system; and c) identify aspects of care for T2DM patients that are in need of improvement. Our work highlights the potential of routine EHR data to contribute to chronic care quality improvement, as well as the need for attention to the quality and completeness of electronic data. These findings are important in the global context of rapidly increasing prevalence of chronic conditions, particularly in low and middle-income countries, which require regular monitoring of processes and outcomes of care to ensure wise use of scarce resources. They also highlight the potential but less than optimal uses of electronic health information systems for routine monitoring of care processes and outcomes [22,23].

### A) QCIs in the context of a middle-income country health care system

Most published QCIs have been constructed for healthcare systems in high-income countries. Using the same QCIs across countries would facilitate international comparisons [24]. However, QCIs should be adjusted according to national variations in financing, structure, and provision of healthcare services, and differences in the characteristics of populations across countries. We originally intended to adopt the HEDIS indicators [18] used by more than 90% of America's health plans to measure performance of care and services. However, since HbA1c, LDL-cholesterol tests and nephropathy screening are not established standards at IMSS family medicine clinics, we needed to develop contextually suitable indicators. Our process for developing, measuring, validating, and reporting QC indicators may be informative in other developing countries.

### B) measuring QCIs using routinely collected data from a widely implemented EHR system

The EHR did not contain all data elements needed to identify the denominator population of members with diabetes or to construct the identified QCIs. Clinical services at IMSS’ FMCs are almost paperless; all information is electronically stored. Our results indicate that there are shortcomings in the electronic information, possibly related to flaws in its design and the limited use of some EHR fields. While we generated rules and algorithms to identify plausible values and exclude records with missing values from the analysis, it is crucial for IMSS to improve its electronic record system for quality monitoring to become a routine process. This would include maintaining a reliable registry of patients with important chronic illnesses like diabetes.

We used a conservative approach to manage missing data, by not including cases with missing information in the analysis. This decision was supported by results from comparisons of socio-demographic characteristics of patients with and without laboratory results, which did not have statistically significant differences; we also took into account that the size of the sample was large, thus reducing the number of cases would not reduce statistical power. However, the validity and generalizability of our results are limited to the extent that there was bias in exclusion of members without visits during the observation year or without data needed to measure QCIs.

To progress to a robust eHealth system, it will be necessary to analyze in-depth the functionality and quality of the EHR, develop decision-support systems, and strengthen linkages between the EHR and other electronic applications, such as laboratory and pharmacy databases.

### C) identifying aspects of diabetes care in need of improvement

Complex healthcare institutions require ongoing evaluation of their performance. In Mexico, evaluating QC based on EHR data is novel and promising, given the high cost, lengthy process, and lack of scale of more typical paper-based evaluations. The lack of complete data notwithstanding, we identified important aspects of care that require improvement. IMSS clinics have low rates of use of diagnostic tests, including HbA1c, microalbuminuria screening and LDL-cholesterol. IMSS’ current model of care relies heavily on family medicine doctors. As our results show, patients have limited access to dietitians, social workers, nurses and other specialists, hindering the integrated multi-specialty care patients with chronic conditions require to ensure the best possible outcomes [25,26].

Our results are comparable to those from studies within Mexico and elsewhere. In a study conducted in Mexico, 66.3% of T2DM patients did not have good metabolic control, although those with access to healthcare had better health outcomes. The European Core Indicators Diabetes Project included 19 countries [27]; its results showed wide variability in QCIs among them: HbA1c testing ranged from 51% (Ireland) to 99% (France and the Netherlands); lipid measurements from 45% (Ireland) to 99% (the Netherlands); and eye examinations from 12% (Ireland) to 84% (The Netherlands). In India, only 13% of T2DM patients had at least one HbA1c measurement, 16.2% had an eye examination, 3.1% foot examination, 8.3% nutritional counseling, 32.1% serum cholesterol estimation and 17.5% were prescribed aspirin; [28]. Rates of HbA1c > 7% varied from 32% (Ireland) to 83% (Cyprus); total cholesterol > 5 mmol/l ranged from 14% (Ireland) to 68% (Cyprus); blood pressure > 140/90 mmHg, from 17% (France) to 46% (Sweden). [27] The QUALIDIAB network [29] in Argentina, Brazil, Chile, Colombia, Paraguay, and Uruguay reported that 43% of patients had blood glucose level of <7.7 mmol/L and the proportion of patients reaching combined treatment goals for blood glucose, cholesterol and blood pressure ranged from 1% in Mexico to almost 12% in the United States [30]. These variations should give rise to continued assessment and improvement efforts.

While this study included information from only four clinics, we found important differences between them; for example, two clinics did not have HbA1c data, pointing to heterogeneity in care processes even within the same health delivery system. Further research studies should examine ways to reduce these differences. Although the study clinics may not represent the QC that IMSS provides, results from desk-based medical chart reviews (with smaller sample sizes) are similar [4,5].

Our results will serve as input to subsequent improvements of the IMSS EHR. The health system in which the EHR is used has a heavy clinical workload (20–25 patients per doctor per 6 hours of consultations, or 15–18 minutes per patient). In this context, the EHR can either hinder or facilitate care. The EHR was originally designed to gather routine clinical information to facilitate the daily work of family doctors and yield epidemiological information. Our study supports the notion that data from the EHR can be used to evaluate quality of care. Further, the EHR could potentially serve other purposes, such as a decision support system to guide clinicians through gathering relevant patient information, including risk factors and health status, and provide drug dosing guidelines and support tools. However the IMSS EHR does not yet include decision support systems. These should be developed as part of the advancement of the EHR. The experience of Mexico in the use of the EHR to evaluate quality of care can be of value for other low and middle-income countries that aim to improve their e-health capabilities.

## Conclusions

This study demonstrated that it was feasible to evaluate QC for T2DM patients by using EHR data. Evaluating QC using EHR information can identify the performance of individual clinics or individual providers, guide future interventions aimed at improving QC and evaluate whether these interventions achieve their expected aims.

A well designed EHR, which should include rigorous data-entry and data quality monitoring would improve the capabilities to evaluate and monitor processes and outcomes of care, which in turn would provide useful feedback to authorities, healthcare personnel and patients. It also can serve to make comparisons among clinics, health care systems or countries.

From the clinicians’ perspective, EHR design and use should improve its functionality for the daily work, for example as a clinical decision-making tool, and as a reliable source to provide feedback to the providers.

## Competing interests

The authors declare that they have no competing interests.

## Authors’ contributions

RPC conceptualized and designed the study, coordinated the fieldwork and wrote the article. SVD conducted the literature review, coordinated the development, definitions, and programming of the indicators, conducted the statistical analysis, and interpreted the data and contributed to drafting the article. MSO programmed the indicators and collaborated on the analysis; ML participated in assessing data quality and programming the indicators and reviewed critically the results and paper. AHP participated in the development of the study. JE and FEL participated in the design of the indicators and reviewed the paper for significant intellectual content. DR-D collaborated on conceptualizing and designing the study, participated in the development of the study and critically reviewed the paper. AKW collaborated on conceptualizing and designing the study, participated in the development of the study, reviewed the results and contributed to drafting the article. All authors approved the final manuscript.

The opinions expressed are those of the authors and do not necessarily represent the views of their institutions.

## Pre-publication history

The pre-publication history for this paper can be accessed here:

http://www.biomedcentral.com/1472-6947/12/50/prepub
